# Supplementary material for: A framework for exhaustively mapping functional missense variants
Source: Mol Syst Biol. 2017 Dec 21;13(12):957. doi: 10.15252/msb.20177908 (PMC5740498; doi:10.15252/msb.20177908)
Supplement: Supplementary file 1 — Appendix [file MSB-13-957-s001.pdf]

A Framework for Exhaustively Mapping Functional Missense Variants - APPENDIX

Table of Contents

Appendix Figures.......... 2
Appendix Tables.......... 13
Appendix Texts.......... 14
Analysis of PopCode's mutational profile.......... 14
Structural and biochemical observations from the UBE2I map.......... 16
Intragenic epistasis and compensatory mutations.......... 19
Structural and biochemical observations in the disease gene maps.......... 21
Appendix Methods.......... 25
UBE2I interface analysis.......... 25
Detection of intragenic epistasis and compensatory mutations.......... 25
Appendix References.......... 26

Appendix Figures

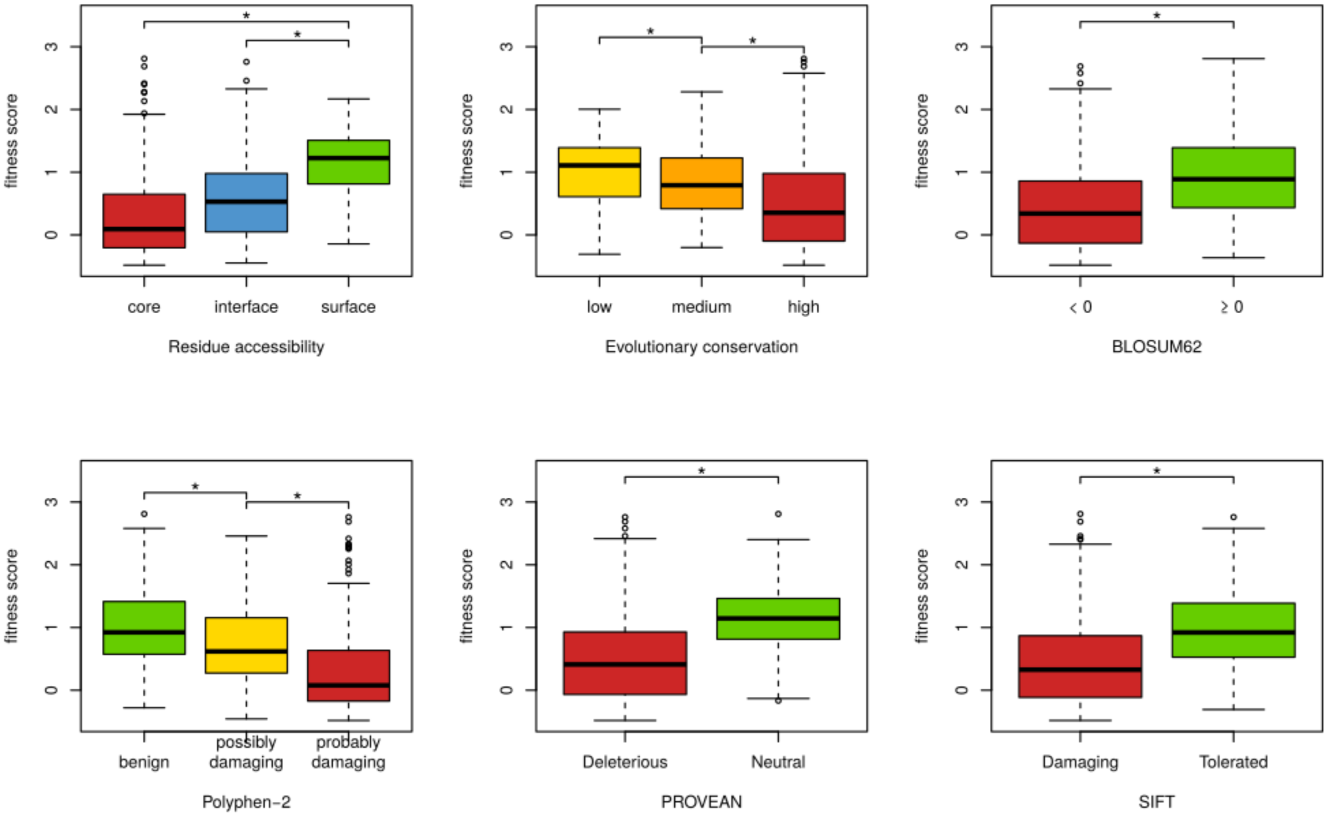

Appendix Figure S1A: BarSeq Complementation screen scores behave as expected when compared to structural context; evolutionary conservation as measured by AMAS; BLOSUM scores; Polyphen-2 predictions; PROVEAN predictions; and SIFT predictions.

One-sided Wilcoxon tests: Core (n=208 clones) vs Surface (n=42 clones) W = 1649, P = 1.01×10^{-10}; Interface (n=215 clones) vs Surface (n=42 clones) W = 2461, P = 1.58×10^{-6}; Low conservation (n=60 clones) vs Medium Conservation (n=105 clones) W = 3789, P = 0.015; Medium Conservation (n=105 clones) vs High Conservation (n=404 clones) W = 28043, P = 1.8×10^{-7}; BLOSUM <0 (n=361 clones) vs BLOSUM >= 0 (n=208 clones) W = 22355, P = 4.4×10^{-16}; Benign (n=190 clones) vs Possibly Damaging (n=107 clones) W = 12648, P = 2.38×10^{-4}; Possibly Damaging (n=107 clones) vs Probably Damaging (n=272 clones) W = 20306, P = 1.03×10^{-9}; Deleterious (n=468 clones) vs Neutral (n=101 clones) W = 10662, P < 2.2×10^{-16}; Damaging (n=390 clones) vs Tolerated (n=179 clones) W = 20053, P = 3.47×10^{-16}

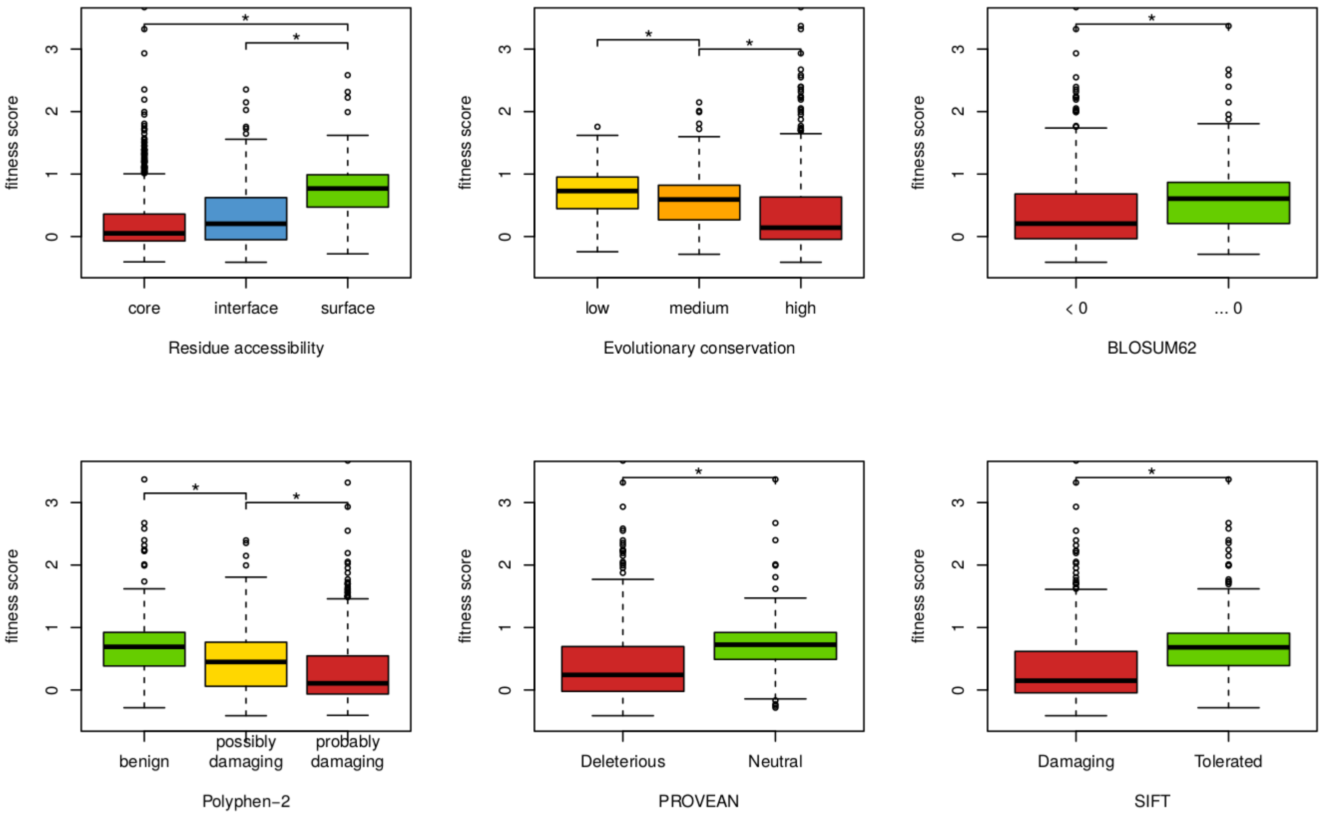

Appendix Figure S1B: TileSeq Complementation screen scores behave the same as BarSeq. One-sided Wilcoxon tests: Core (n=1013 clones) vs Surface (n=233 clones) W = 44034, P < $2.2x10^{-16}$; Interface (n=1099 clones) vs Surface (n=233 clones) W = 59892, P < $2.2x10^{-16}$; Low conservation (n=263 clones) vs Medium Conservation (n=563 clones) W = 81136, P = $3.3x10^{-7}$; Medium Conservation (n=563 clones) vs High Conservation (n=1915 clones) W = 618833, P < $2.2x10^{-16}$; BLOSUM <0 (n=2076 clones) vs BLOSUM >= 0 (n=665 clones) W = 445867, P < $2.2x10^{-16}$; Benign (n=664 clones) vs Possibly Damaging (n=492 clones) W = 180761, P = $3.6x10^{-13}$; Possibly Damaging (n=492 clones) vs Probably Damaging (n=1585 clones) W = 413069, P < $2.2x10^{-16}$; Deleterious (n=2428 clones) vs Neutral (n=313 clones) W = 185654, P < $2.2x10^{-16}$; Damaging (n=2031 clones) vs Tolerated (n=710 clones) W = 352852, P < $2.2x10^{-16}$

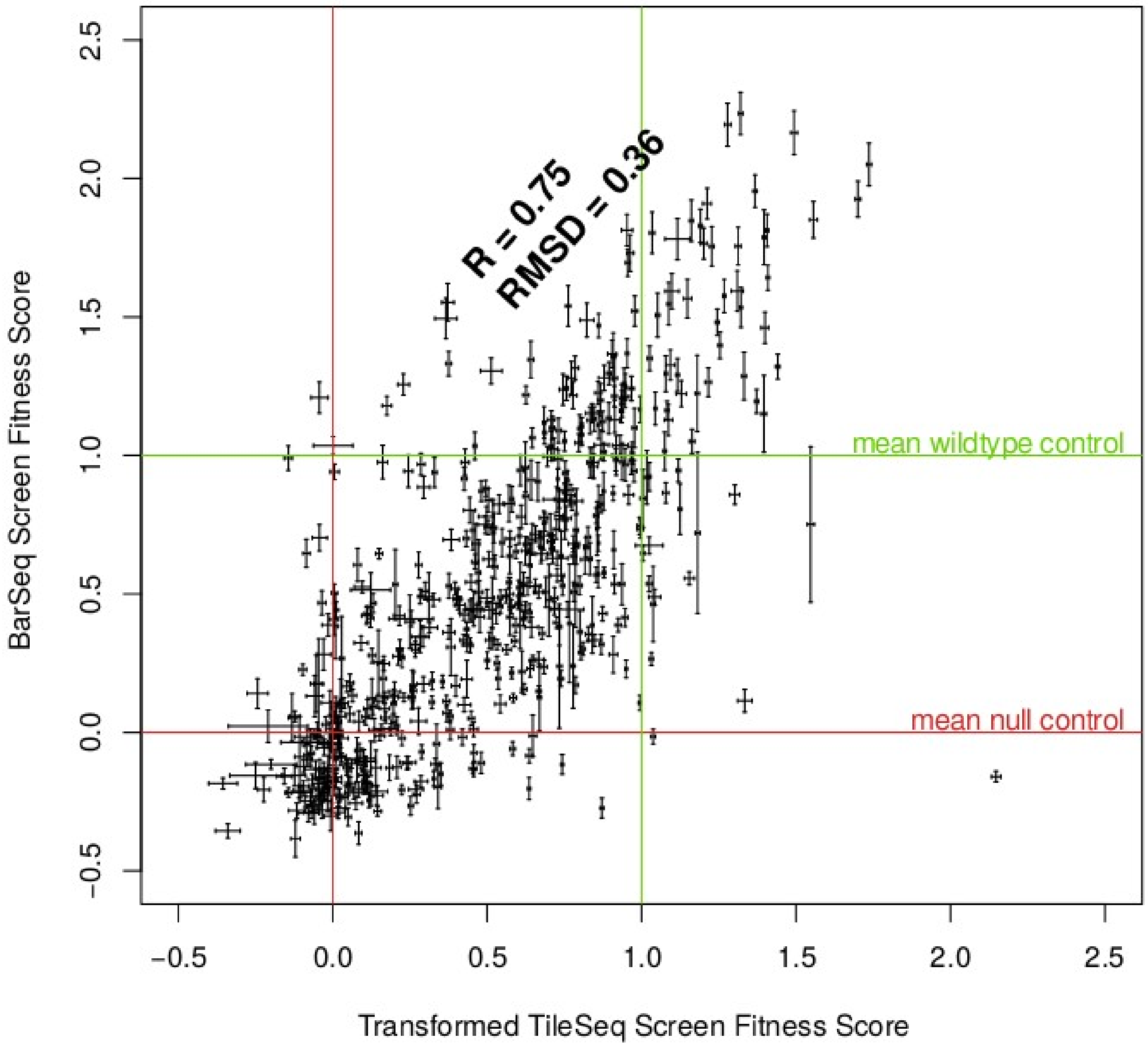

Appendix Figure S2: Correlation between DMS-BarSEQ and DMS-TileSEQ complementation scores for UBE2I. Error bars: Bayesian regularized s.e.m.

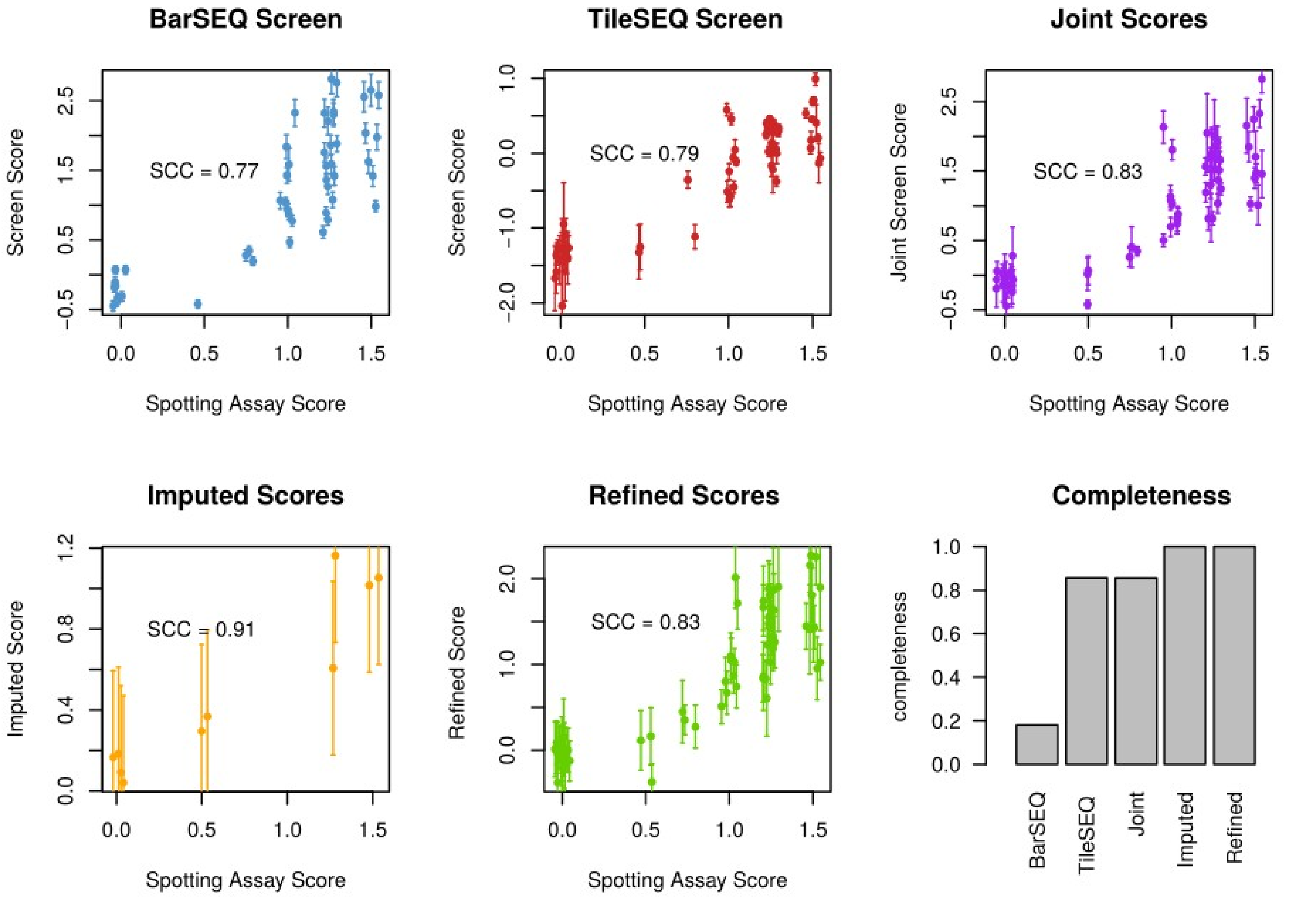

Appendix Figure S3: Spearman correlation of DMS complementation scores with manual complementation spotting assays at different stages of data processing (all panels except bottom right) Error bars for BarSeq, TileSeq, Joint and Refined scores show Bayesian regularized s.e.m; error bars for Imputed scores show RMSD in lieu of s.e.m. Jitter was added to the x-axis for clarity. See methods for quantification of spotting assays.

Bottom right panel: map completeness i.e. the fraction of missense variants with a complementation score passing the quality filter (see Materials and Methods).

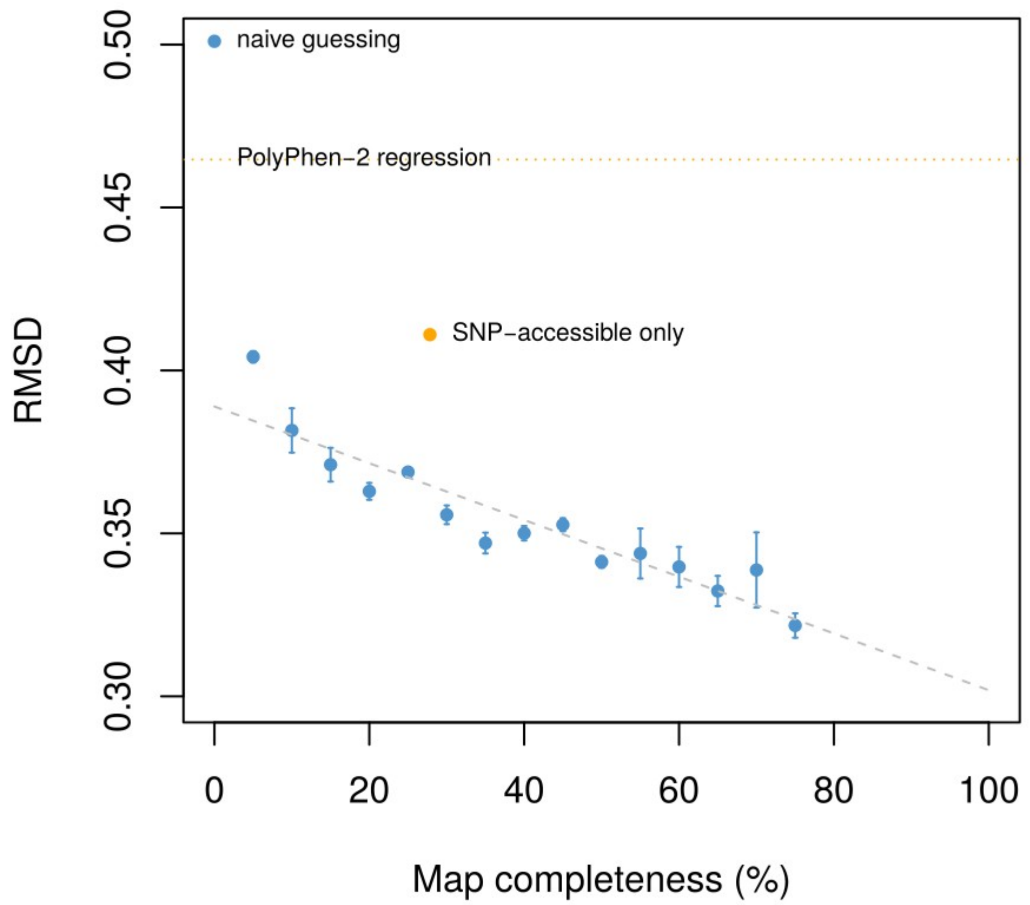

Appendix Figure S4: Subsampling analysis of the imputation method. The impact of training set size on imputation RMSD. The datapoint at sample size 0 represents naive guessing, i.e. using the score average as the prediction for every variant. Training only on SNP-accessible variants (orange dot) yields significantly worse RMSD than using random variants across the full amino acid spectrum with the same sample size. For reference, the performance of simple regression on PolyPhen-2 scores is shown as well.

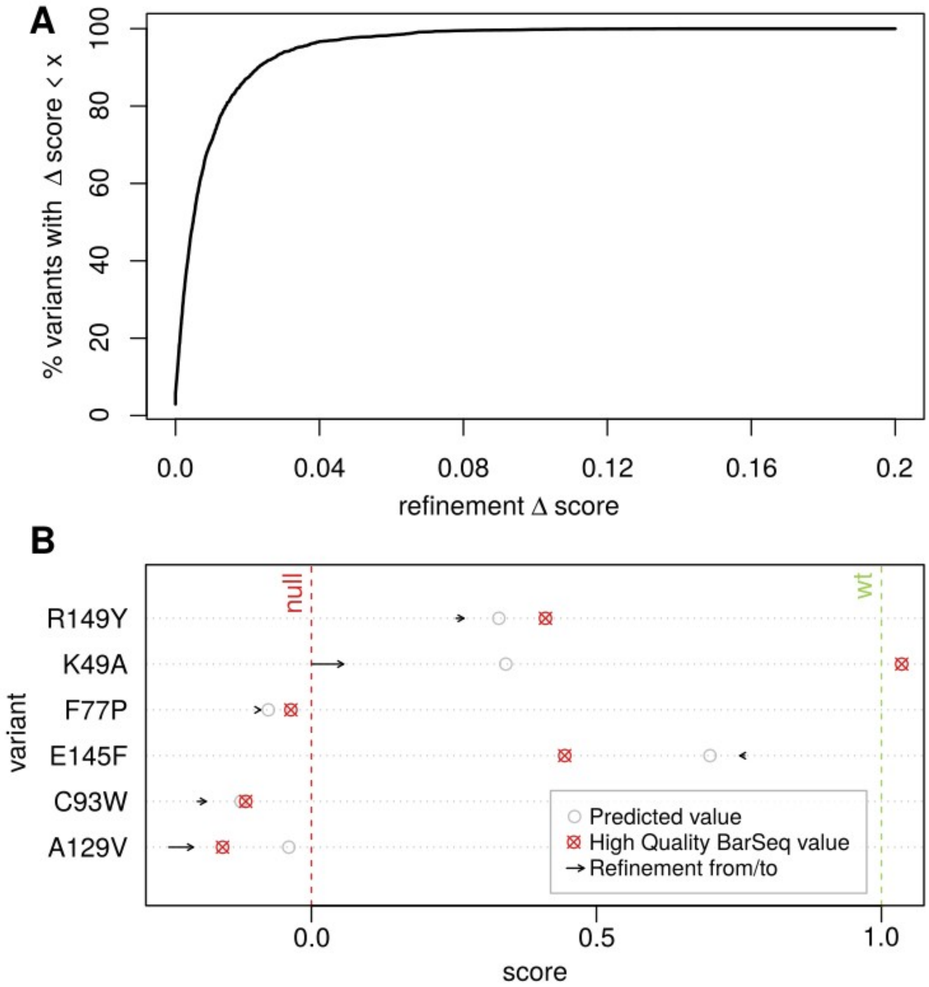

Appendix Figure S5: Effects of refinement. A) Cumulative distribution of changes to the fitness scores on the map as a result of regularization. B) Six variants were found that were well measured in DMS-BarSeq but less well measured in DMS-TileSeq. For evaluation, regularization was performed only on the DMS-TileSeq data and compared to the DMS-BarSeq gold standard. Base and tip of arrows indicate pre- and post-regularization values, gray circles indicate the machine learning predictions used. Red targets indicate BarSeq gold standard.

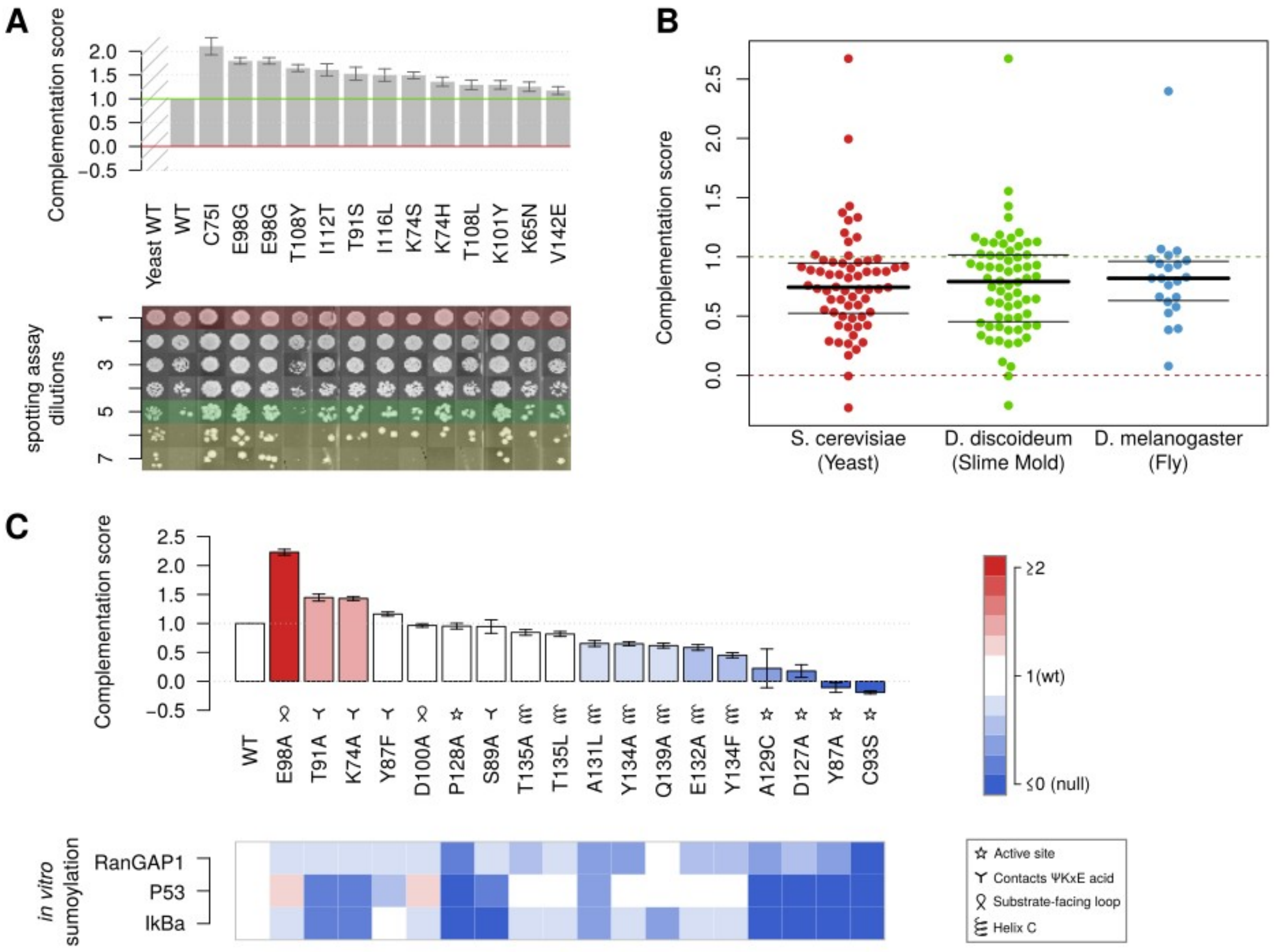

Appendix Figure S6: A) Spotting assay validation of above-WT complementation. Bar height represents refined scores. Error bars show Bayesian regularized s.e.m. Green lane: Maximum dilution step at which human wt is visible. Yellow lanes: Dilution steps above human wt visibility. B) Comparison of fitness scores for reversions to yeast residues compared to amino acid changes naturally occurring in slime mold and fly. C) Comparison with in vitro sumoylation results reveal substrate specificity shifts. Error bars: Bayesian regularized s.e.m.

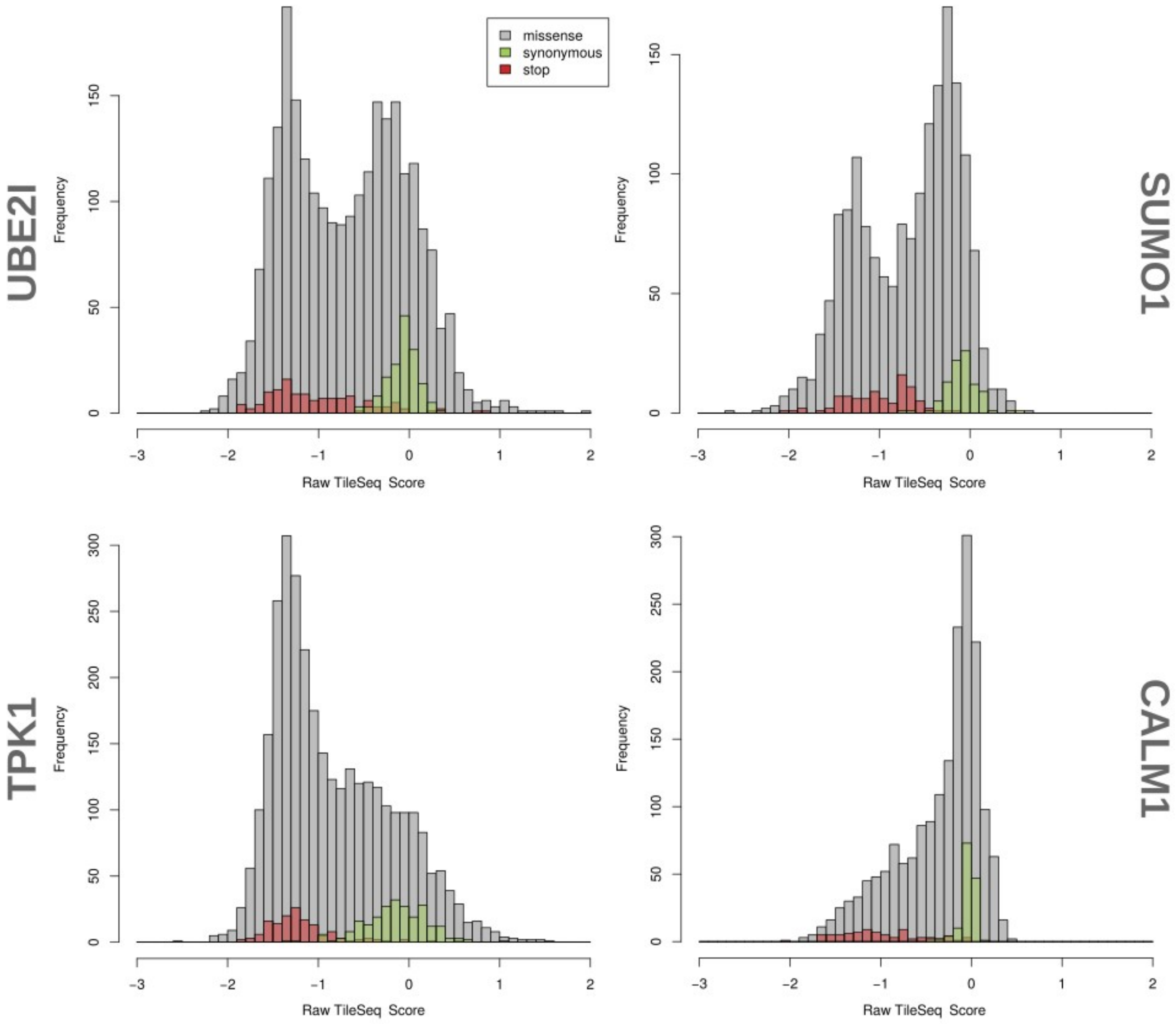

Appendix Figure S7: Distributions of raw DMS-TileSeq library scores for missense, synonymous and stop mutations for all four variant maps. The raw TileSeq score is based on the ratio between the allele frequencies in the selection condition and the non-selection conditions.

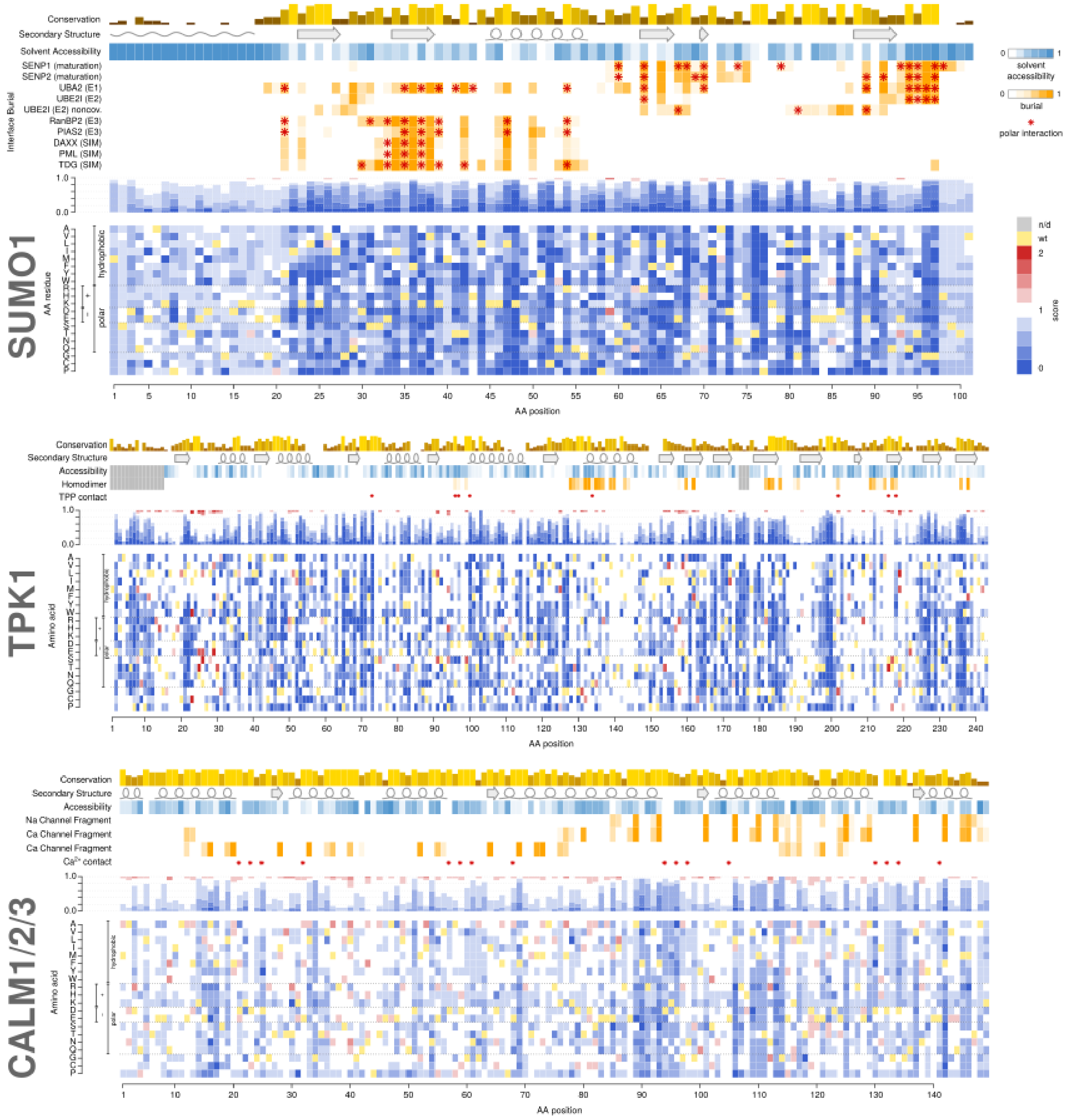

Appendix Figure S8: Untransformed maps of SUMO1, TPK1, and Calmodulin. Colors as in Main Figure 3

A

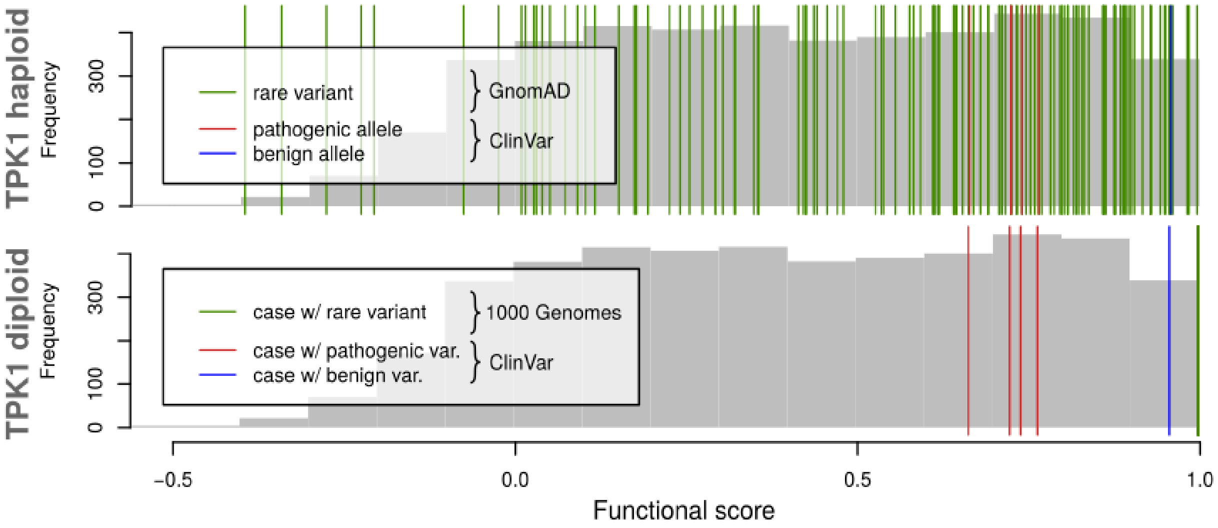

B

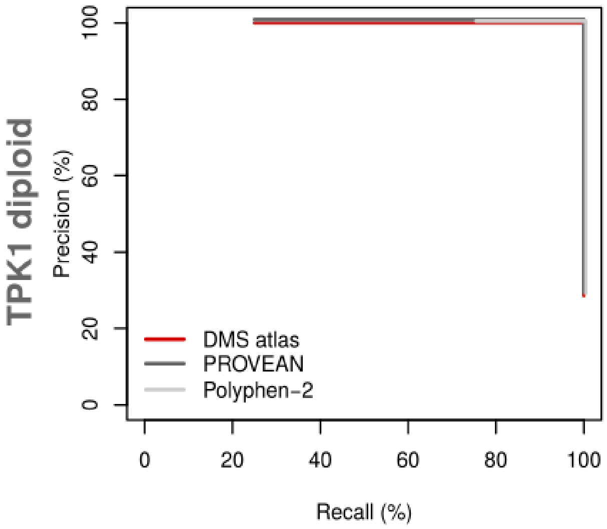

Appendix Figure S9: A) Refined and imputed functional scores for TPK1 variants. Top panel: Variants from GnomAD and Clinvar evaluated in a virtual haploid context, i.e. not accounting for the recessive nature of the locus. Bottom panel: Scores for individual cases from 1000 Genomes and ClinVar taking into account the diploid genotypes and recessive nature of the locus. B) Precision-Recall plots for the DMS atlas, PROVEAN and PolyPhen-2 when adjusting TPK1 predictions to diploid genotypes.

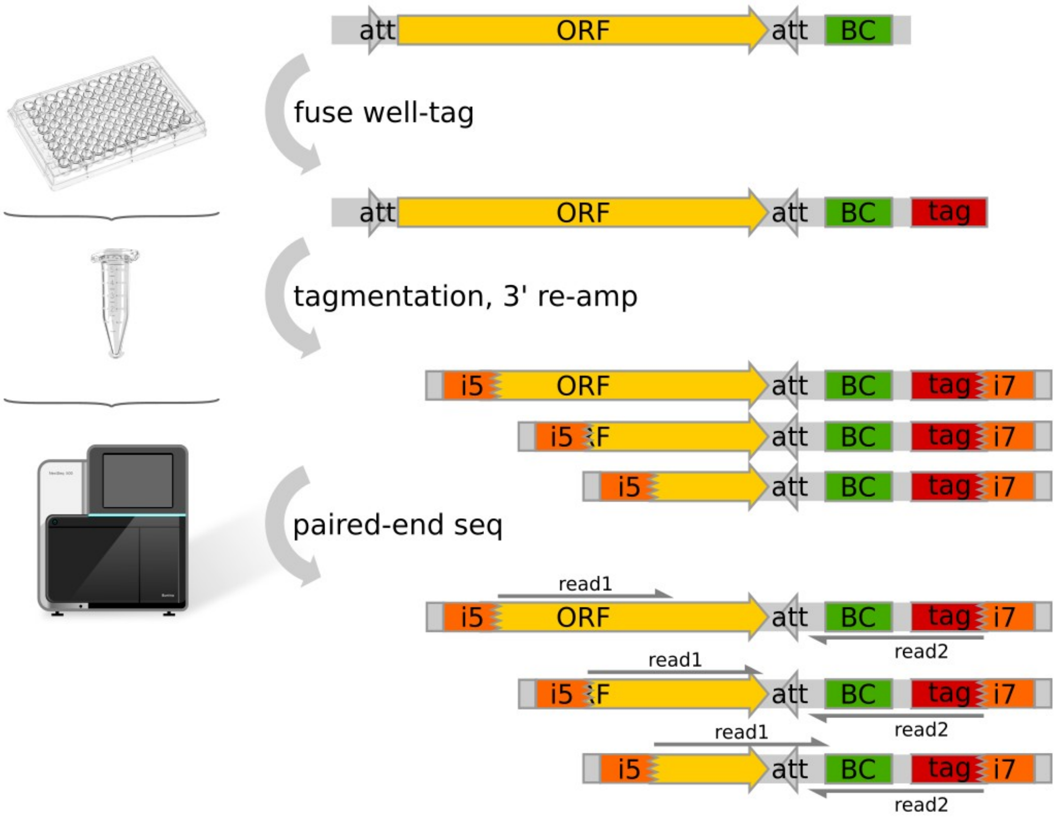

Appendix Figure S10: KiloSeq. 1) For each library well, amplicons containing the variant ORF (gold) and Barcode locus (green) are amplified with primers adding a well-specific tag. 2) Tn5 tagmentation fragments the DNA while simultaneously adding Illumina i5/i7 linkers. 3' re-amplification enriches for fragments containing the well tags. 3) Each pair of sequencing reads now contains a fragment of ORF sequence and the associated barcode and well tag.

Appendix Tables

Expanded View dataset EV1: Overview of previous DMS studies, their underlying selection methods, sample space, completeness and quality control methods.

See separate spreadsheet file.

Expanded View dataset EV2: See separate spreadsheet file.

Appendix Table S1: Comparing different models for the effects of activity-enhancing mutations

**Protein**	**Model**	**ΔAIC relative to best model**
UBE2I	Penalize enhancing mutations	0
UBE2I	Cap score of enhancing mutations at wildtype level	27.7
UBE2I	Enhancing mutations beneficial	60.6
SUMO1	Penalize enhancing mutations	0
SUMO1	Cap score of enhancing mutations at wildtype level	23.9
SUMO1	Enhancing mutations beneficial	52

Appendix Table S2: Practical comparison of DMS-BarSeq and DMS-TileSeq

	DMS-BarSeq	DMS-TileSeq
Arrayed Library	X	
Requires Robotics	X	
KiloSeq	X	
Multi-mutant clone readouts	X	
Epistasis analysis	X	Limited
Direct clone-specific-readout	X	
Susceptible to confounding		X

Appendix Texts

Analysis of PopCode's mutational profile

Over half of the clones in the library conformed to BarSeq quality criteria: (i) They contained at least one missense mutation, (ii) did not contain any insertions or deletions, (iii) did not contain mutations outside of the ORF, (iii) had unique barcodes, and (iv) had sufficient read coverage during KiloSeq to allow for confident genotyping. The single largest reason for exclusion was the occurrence of indels and CNVs (Appendix Figure ST1A). An analysis of the mutation signatures across clones generated by POPCode revealed that two different mechanisms appear to underlie mutagenesis. When considering only mutations that change more than one base in a given codon, there is an equal chance for every possible base except in the third position, where almost no adenine or cytosine was introduced. This is consistent with the NNK degeneracy code used in the POPCode oligo design. By contrast, variants that change only a single base in a given codon show a strong bias for transitions over transversions. These could be introduced due to polymerase error (Appendix Figure ST1B). This secondary source of variation is also reflected in the relative share of single nucleotide variants, which make up 56\% of mutations (Appendix Figure ST1C). As a consequence, when examining the mutation coverage across the sequence of the ORF, it is clearly visible that the share of amino acids reachable with a single nucleotide change from the respective wildtype codon is much closer to saturation than the the set of all possible amino acid changes (Appendix Figure ST1D). Additionally, some hotspots are visible in which the mutation rate is higher, which is likely due to different hybridization efficiencies of oligos across the ORF sequence.

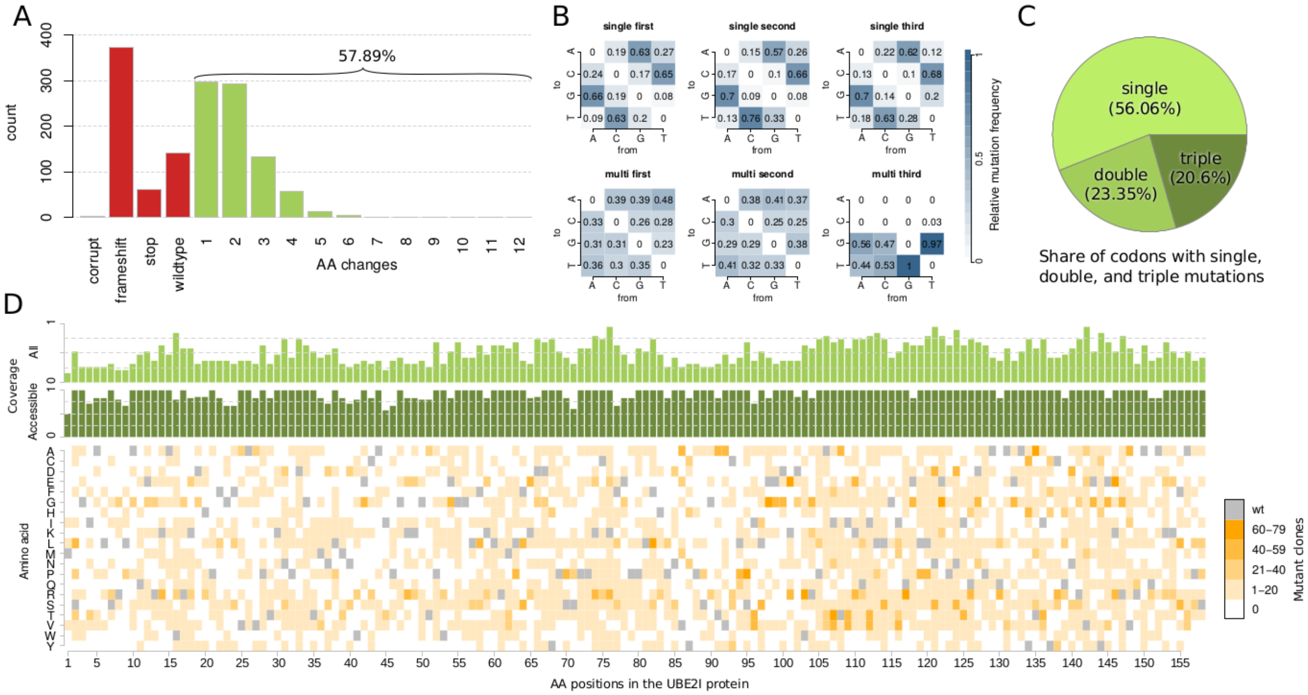

Appendix Figure ST1: KiloSeq-based census of the UBE2I POPCode library. A) Breakdown of KiloSeq results for a set of five 384 well plates of mutant clones generated by POPCode. Corrupt: Clones containing mutations outside of the ORF; Frameshift: Clones containing indels or copy number variants; Stop: Clones containing stop codons. B) Breakdown of mutations in codons. Top: Single nucleotide variants; Bottom: Multi-nucleotide variants. Columns correspond to the first, second and third position in a codon. C) Relative shares of single, double and triple nucleotide variants among all missense variants in the library. D) Coverage map of missense variants in the library. Light green track: Coverage across all possible amino acids; Dark green track: Coverage across amino acids reachable with a single nucleotide change from the wildtype codon.

Structural and biochemical observations from the UBE2I map

Based on the DMS map of UBE2I (Figure 2A) several biochemical observations could be made. Consistent with observations made in smaller-scale biochemical studies of the SUMO E2 conjugase (Bencsath *et al*, 2002; Bernier-Villamor *et al*, 2002), the areas most sensitive to mutation are those proximal to the active site (particularly residues 81-88, 90, 92-96, and 127-130), and the N-terminal alpha helix which mediates four protein interactions including the critical interaction with the E1 SUMO-activating complex. Another interesting feature of the map is the alternating tendency towards damaging and benign substitutions across positions 55-65. A comparison with solvent accessibility reveals this to be caused by alternating externally and internally-oriented residues, with the latter positions constrained to be hydrophobic. This alternating tendency is also reflected in evolutionary conservation across these positions.

When comparing individual protein interaction interfaces, the most substantial fitness defects are observed in the interfaces for the E1 activating complex binding interface and the covalent and noncovalent SUMO binding interfaces (Appendix Figure ST2). The importance of residues at the interfaces mediating covalent and noncovalent interaction SUMO is made apparent by coloring the surface of the UBE2I structure with the median fitness score at each residue position (Figure 2B).

Interestingly, residues A15 and T108 appear adaptive but do not face towards the substrate, instead forming part of the interface with the E3 SUMO ligase RanBP2, and flank a small cavity on UBE2I's surface into which RanBP2 inserts a phenylalanine residue upon binding. Changing either A15 or T108 into aromatic residues results in a large fitness increase, which may result from gain of a π-stack interaction that strengthens E2-E3 binding (Appendix Figure ST3A).

Another interesting observation can be made with respect to a known phosphorylation site on the surface of UBE2I. Su and colleagues previously discovered that phosphorylation of Serine 71 via the Cyclin-dependent Kinase CDK1 results in sumoylation hyperactivity (Su *et al*, 2012). Our map shows that substitutions with phosphomimetic residues at this position lead to hyperactive complementation, consistent with Su et al.'s observations. Furthermore, other residues amenable to phosphorylation are also tolerated, while hydrophobic replacements are generally deleterious (Appendix Figure ST3B)

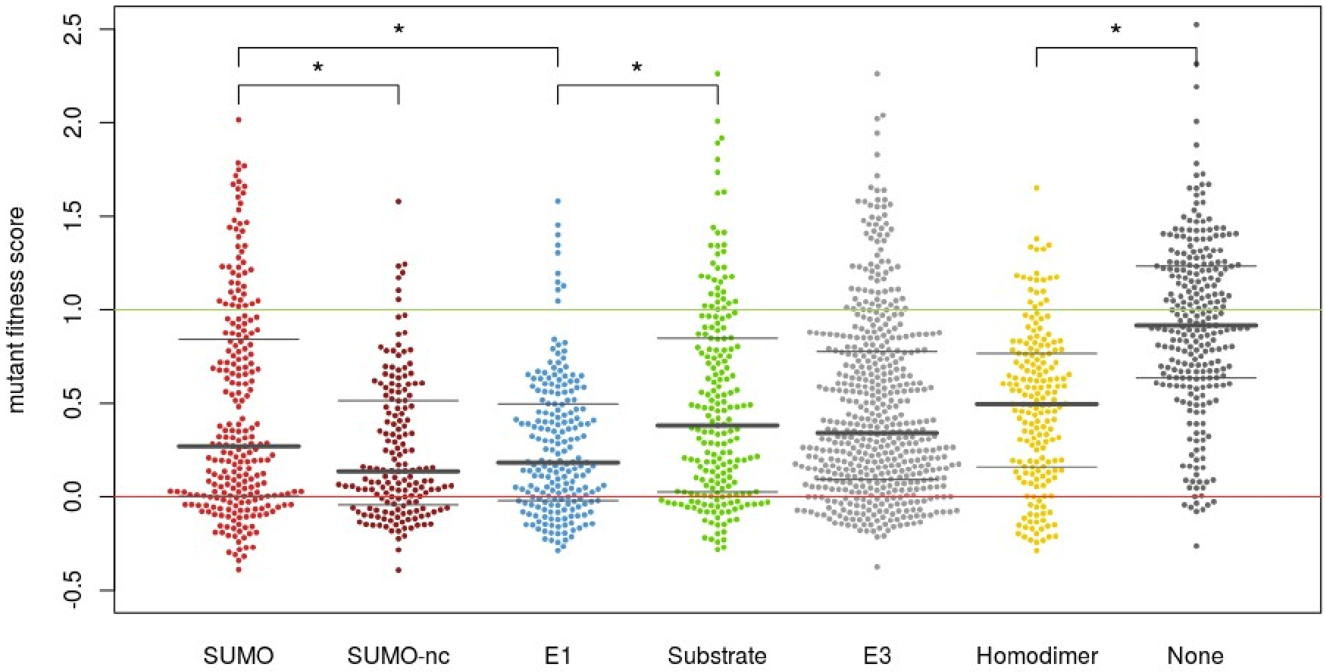

Appendix Figure ST2: Regularized and imputed score distributions reveal relative importance of protein interaction interfaces. SUMO: Covalent SUMO interface; SUMO-nc: non-covalent SUMO interface; E1: E1 activating complex interface; Substrate: RanGAP1 binding interface; E3: RanBP2 binding interface; Homodimer: UBE2I Homodimerization interface; None: Surface residues not involved in any interface.

Wilcoxon rank sum tests with continuity correction: Sample sizes (unit:variants): SUMO:247, SUMO-nc:171, E1:209, Substrate:209, E3:513, Homodimer:190, None:266; alt.hyp.= SUMO > SUMO-nc, W = 24909, p-value = 0.0009016; alt.hyp.= SUMO-nc < E1, W = 17133, p-value = 0.2448; alt.hyp.= SUMO > E1, W = 29508, p-value = 0.004195; alt.hyp.= Substrate > E1, W = 26845, p-value = 2.541e-05; alt.hyp.= None > Homodimer, W = 37982, p-value < 2.2e-16; alt.hyp.= Homodimer > E1, W = 26017.5, p-value = 4.256e-08; alt.hyp.= Homodimer > E3, W = 52120, p-value = 0.07848; alt.hyp.= Homodimer > SUMO-nc, W = 21596, p-value = 3.249e-08

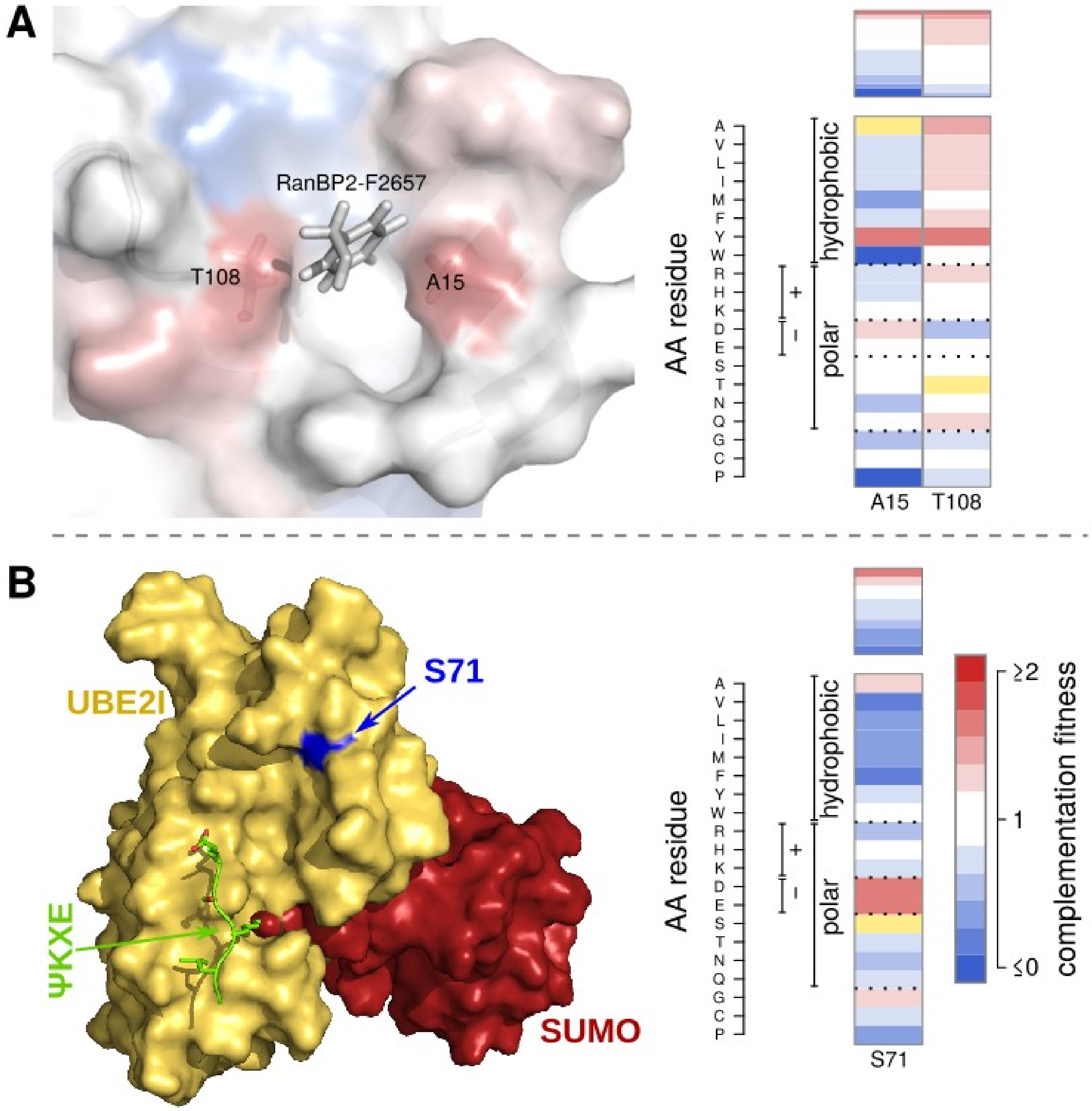

Appendix Figure ST3: (A) Potential de-novo pi-stack interaction strengthening E2-E3 binding.

(B) A known phosphorylation site at S71 shows hyperactive complementation when replaced with phosphomimetic residues.

Intragenic epistasis and compensatory mutations

Full-length UBE2I clones generated for DMS-BarSeq analysis often encoded more than one amino acid change. Multi-mutant clones offer the opportunity to search for intragenic genetic interactions. Genetic interaction is defined when a combination of mutations yields an unexpected phenotypic effect, so that identifying genetic interactions requires that we model the phenotype expected from a combination of mutations, given the single-mutant effects. Here we used a previously-described multiplicative model (Phillips, 1998; St Onge *et al*, 2007) in which genetic interaction is measured as $\varepsilon_{ij}=f_{ij}-f_i \cdot f_j$, where f_i and f_j represent single mutant fitness and f_{ij} represents double mutant fitness scores. Most double mutants (803 of 1132, or 71%) did not show a significant deviation from $\varepsilon_{ij}= 0$ under this model, while 328 position pairs did show significant genetic interaction (Appendix Figure ST4A). Of particular interest are compensatory interactions, i.e. cases where a double mutation is more fit than either of the component single mutations. Where compensatory residues are proximal in the protein structure, the combination of two mutant residues may be able to re-establish a physical interaction that was lost in each of the single mutants. Although the majority of genetically interacting sites were not proximal in the structure (Appendix Figure ST4B), there were interesting exceptions. For example, the I4T-P69S double mutant appears to exhibit compensatory behaviour: In the wild type structure, the van-der-Waals radii of the two residues are in direct contact (Appendix Figure ST4C). Either mutation alone would be expected to destabilize the hydrophobic interaction between isoleucine and proline. However, In the double mutant, hydroxyl groups on the two residues could adopt a hydrogen bond that re-establishes interaction and re-stabilizes the fold (Appendix Figure ST4D/E).

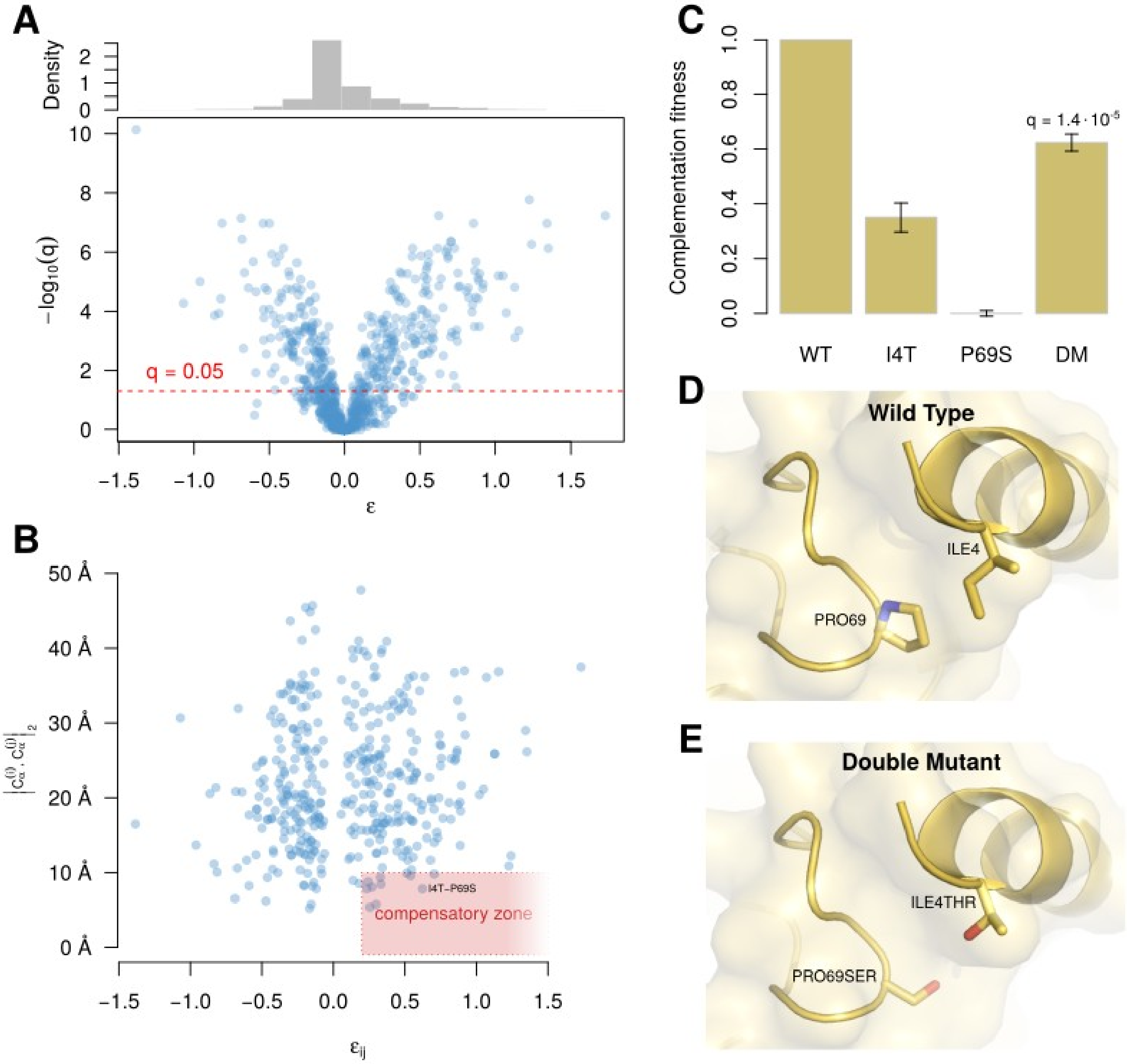

Figure ST4: Intragenic epistasis and compensatory mutations in UBE2I. (A) Volcano plot of genetic interaction strength and associated FDR q-values. (B) Significant genetic interaction values compared to euclidean distance of involved amino acid alpha carbon atoms in the UBE2I structure. Double mutants with positive genetic interactions within 10Å of each other are considered candidates for compensatory mutations. (C) Impact scores for ILE4THR and PRO69SER indicate a strongly positive genetic interaction. (D) Detail of UBE2I crystal structure. ILE4 and PRO69 contact each others' VDW radii. (E) Same as D, with a simulated double mutant.

Structural and biochemical observations in the disease gene maps

<u>SUMO1:</u> The SUMO1 map displayed a strong enrichment for neutral substitutions within the first 20 amino acid positions (Figure 4, top panel), which is consistent both with the low level of evolutionary conservation for this region and its annotation as a disordered region. The last four amino acid positions were similarly insensitive to mutation, consistent with removal of this region by SENP proteases during maturation. By contrast, other residue positions were strongly sensitive to mutation, including many inward-facing residues that are apparently constrained to be hydrophobic. As expected, the C-terminal diglycine of mature SUMO, which is required for covalent binding of SUMO to the E1, E2 and to the sumoylation target protein, is very sensitive to mutation. Other strongly constrained residues are core members of interaction interfaces. These include the central phenylalanine 36 in the SUMO recognition motif (SRM) interface; glycine 68, which forms the apex of a tight turn within the interface with de-sumoylation enzymes, as well as the E1 and E2 proteins; and leucine 80, which is part of the interface with non-covalently bound E2.

The proximity and orientation of aspartate #73 and lysine #48 suggests that they are able to form a salt bridge with one another. The importance of each residue according to the DMS map supports a model in which this salt bridge is important for SUMO folding and/or stability. Interestingly, substituting aspartate for methionine #59, which points towards lysine #48 from an angle similar to that of aspartate #73, enhances the complementation fitness of SUMO1 beyond wild type levels. This further underlines the potential structural importance of a polar interaction involving lysine #48 (Appendix Figure ST5).

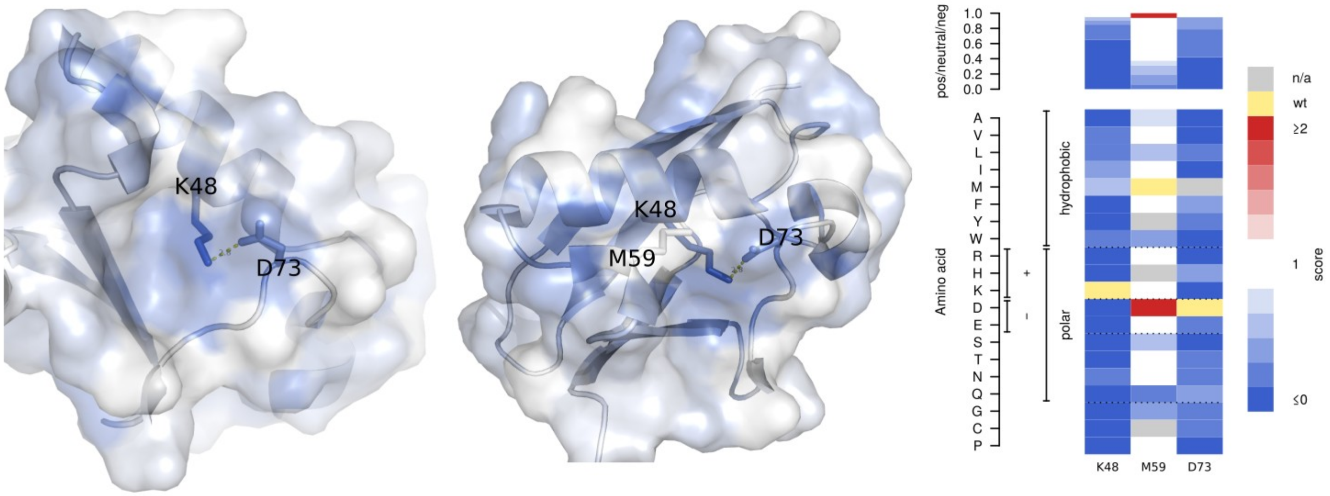

Appendix Figure ST5: A crucial salt bridge within SUMO1

Thiamine pyrophosphokinase 1: TPK1 functions as a dimer, with the dimer interface forming a symmetric pair of active sites that each bind the substrate, thiamine diphosphate (Timm *et al*, 2001) (see Appendix Figure ST6). Each TPK1 monomer is comprised of an N-terminal globular domain and a C-terminal β-sandwich domain. The residues most sensitive to mutation in the protein make up the hydrophobic cores of the two domains: L21, V22, W36, G48, Y53, P65, G70, Y83, L108, I122, T124, and G127 for the N-terminal domain; and L161, G168, G199, L200, V227, V229, L236, and W237 for the C-terminal domain. Mutation-sensitive residues include those closely involved in the active sites: D46, G70, D71, D73, D100, and K103 in the N-terminal half of the active site, contacting the diphosphate portion of the substrate. In the C-terminal half of the active site, K203, L209, G212, L214, S216, T217, and N219 show similar sensitivity. Interestingly, the tryptophan residue at position 202 appears to be insensitive to mutation despite its close and extensive contact with the thiamine ligand. By contrast, a neighbouring lysine at position 201 is surprisingly sensitive suggesting potential importance in coordinating the ligand. Other important dimerization interface residues included M136, G184, V188, G189 and G211. Finally, residues 1-12, which form a beta-strand connecting N- and C-terminal domains, were also found to be sensitive.

CALM1, CALM2 and CALM3: Within Calmodulin, the regions most sensitive to mutation were: 1) the hydrophobic cores of the two globular domains; 2) interfacial residues for CALM1,2,3 protein interactions, and 3) a subset of the negatively charged residues in the EF hands that contact Ca++ ions (Appendix Figure ST7A). Within the hydrophobic cores of the two lobes, five mutually-interacting phenylalanine residues at positions 17, 69, 90, 93, and 142 were among the top 9 most sensitive residues (Appendix Figure ST7B). Within the interaction interface, the residues D85, A89, F93, M100, L106, V109, L113, G114, L117, M125, V137, F142, M145, M146 are the most strongly sensitive to mutation. Regarding the four Calcium-binding EF-hand loops, we found it interesting that only a subset of the negatively-charged residues contacting Ca^{2+} are even moderately sensitive. Within EF1, only D25 appears to be important, in EF2 only N61, in EF3 only D94 and D96, and in EF4 only D130 and D134. Overall, the EF3/4 in the C-lobe also appear to be more important than their N-lobe counterparts. This is in agreement with previous reports that the C lobe is the primary sensory mechanism (Sarhan *et al*, 2012). Some sensitivities are unexplained, e.g. R91 and N54 show strong phenotypes despite extending from seemingly unused surfaces of the protein, potentially pointing to undiscovered functionally sites of interaction or modification.

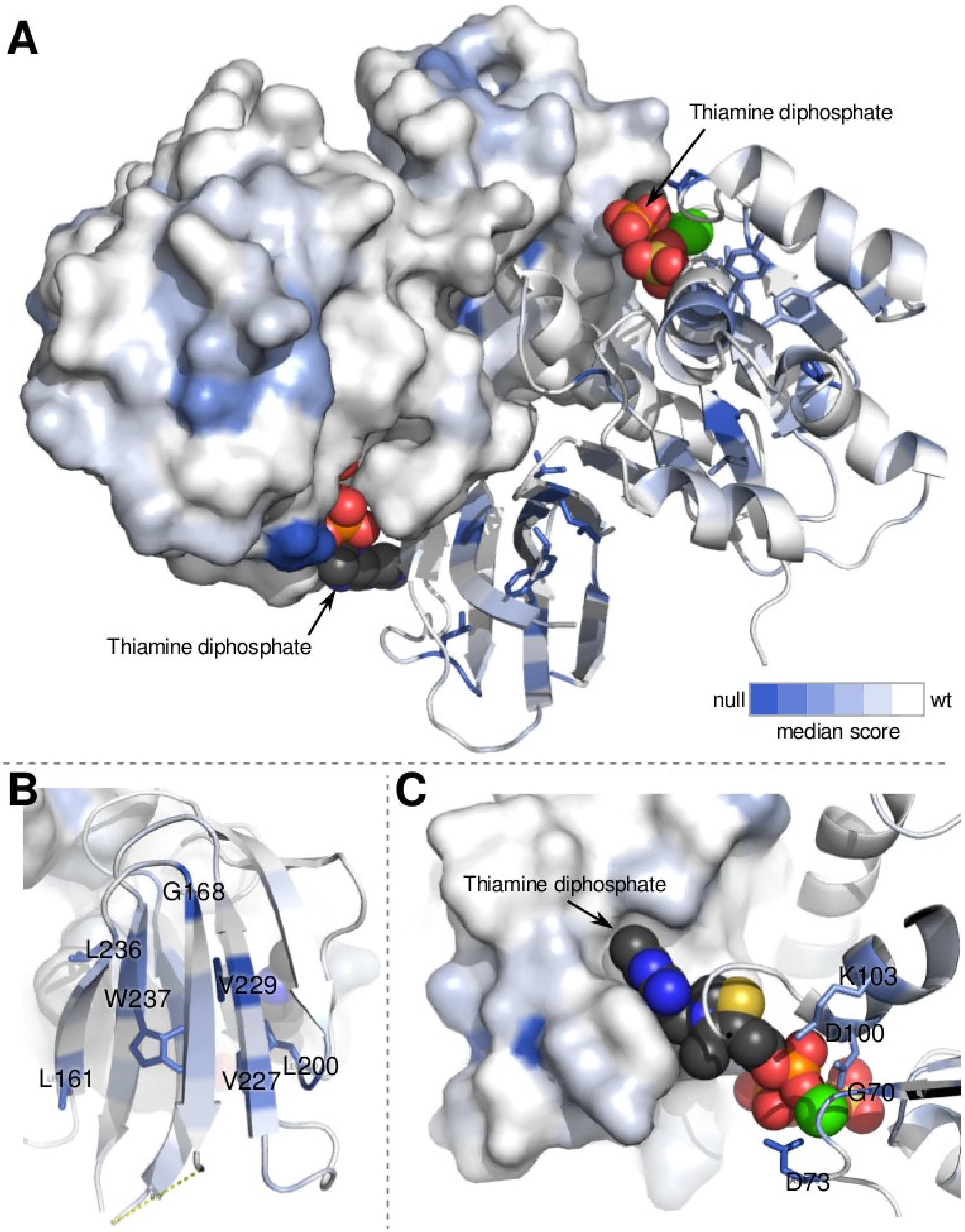

Appendix Figure ST6: Thiamine pyrophosphokinase 1 coloured by median complementation score. A) TPK1 homodimer structure showing one monomer as surface model, the other monomer as cartoon model. B) Hydrophobic residues facing the inside of the C-terminal beta-sandwich domain are sensitive to mutation. C) Active site residues in contact with the substrate are sensitive to mutation. Structure data from PDB:3S4Y

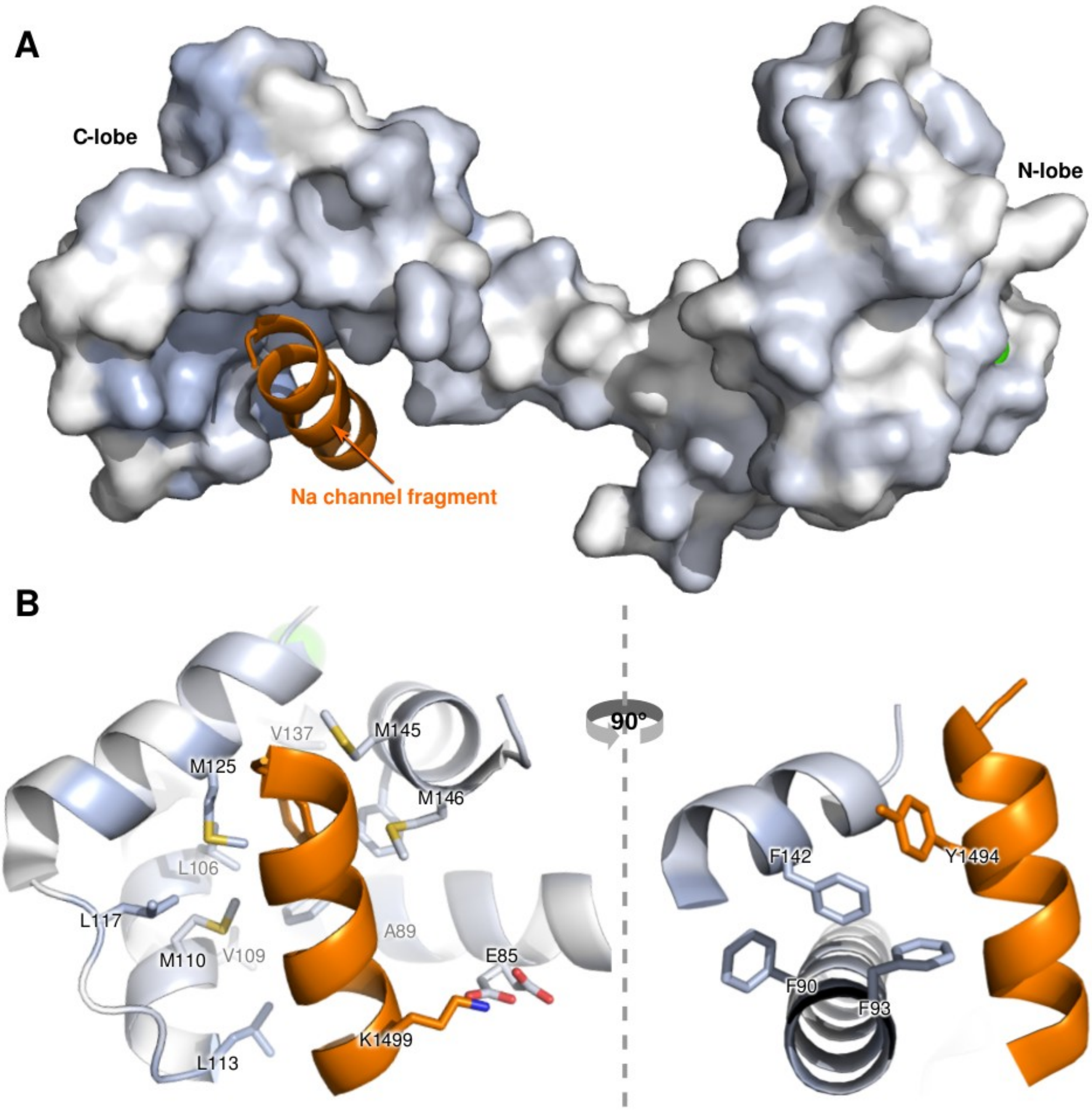

Appendix Figure ST7: Calmodulin coloured by median complementation score. A) Overall structure in complex with with a Na channel fragment. B) Left: Residues at the interaction interface are sensitive to mutation. Right: Phenylalanines 90,93 and 142 are among the most sensitive residues in the protein.

Appendix Methods

UBE2I interface analysis

Co-crystal structure data for UBE2I was obtained from the PDB (Entries: 3UIP; 4W5V; 3KYD; 2UYZ; 4Y1L). A custom script was developed to obtain solvent accessibility using GETAREA (Fraczkiewicz & Braun, 1998) for monomers and complexes, allowing for the calculation of relative burial of interfacial residues. Complementation fitness distributions for each interaction's interfacial residues were tallied and tested for statistically significant differences using Wilcoxon tests. Distributions were plotted using the R package beeswarm (Eklund, 2016). The methods were implemented as part of a larger DMS analysis package available online at http://dalai.mshri.on.ca/~jweile/projects/popcodePipeline/doc/.

Detection of intragenic epistasis and compensatory mutations

One advantage of the barcoded screening approach over regional sequencing is the ability to examine the effects of multiple mutations acting together. To identify simple binary genetic interactions we first validated the standard multiplicative model. We extracted the set of all double mutants in the data, for which the corresponding single mutants were also measured. Defining the genetic interaction effect size as

$$\varepsilon_{ij}=f_{ij}-f_i\cdot f_j,$$

where f_{ij} is the fitness of the double mutant and f_i and f_j are the respective single mutant fitness levels. The distribution of epsilon values forms a bell curve with mode 0.

To find significant deviations from the multiplicative model we calculated the standard deviation of the expected double mutant fitness from the Bayesian regularized standard deviations of the single mutants as follows:

$$V(XY) = E(X^2Y^2) - (E(XY))^2 = V(X)V(Y) + V(X)(E(Y))^2 + V(Y)(E(X))^2$$

This allowed us to perform Student t-tests between the expected and measured double mutant fitness values, which were subsequently corrected for multiple hypothesis testing using the (Benjamini & Hochberg, 1995) method. Double mutants with q values below 0.05 were considered intragenic epistatic relationships.

To generate a list of candidates for compensatory mutation pairs, we integrated the list of genetic interactions with the Euclidian distance between the respective amino acid alpha carbon atoms. Distances were computed using the atomic coordinates provided in PDB structure 3UIP. Positive genetic interactions with effect sizes larger than 0.5 and alpha carbon distance below 10Å were considered candidates for compensatory relationships. The methods were implemented as part of a larger DMS analysis package available online at http://dalai.mshri.on.ca/~jweile/projects/popcodePipeline/doc/

Appendix References

Bencsath KP, Podgorski MS, Pagala VR, Slaughter CA & Schulman BA (2002) Identification of a Multifunctional Binding Site on Ubc9p Required for Smt3p Conjugation. *J. Biol. Chem.* **277:** 47938–47945

Benjamini Y & Hochberg Y (1995) Controlling the False Discovery Rate: A Practical and Powerful Approach to Multiple Testing. *J. R. Stat. Soc. Ser. B Methodol.* **57:** 289–300

Bernier-Villamor V, Sampson DA, Matunis MJ & Lima CD (2002) Structural Basis for E2-Mediated SUMO Conjugation Revealed by a Complex between Ubiquitin-Conjugating Enzyme Ubc9 and RanGAP1. *Cell* **108:** 345–356

Eklund A (2016) The Bee Swarm Plot, an Alternative to Stripchart Available at: https://CRAN.R-project.org/package=beeswarm

Fraczkiewicz R & Braun W (1998) Exact and efficient analytical calculation of the accessible surface areas and their gradients for macromolecules. *J. Comput. Chem.* **19:** 319–333

Phillips PC (1998) The Language of Gene Interaction. *Genetics* **149:** 1167–1171

Sarhan MF, Tung C-C, Petegem FV & Ahern CA (2012) Crystallographic basis for calcium regulation of sodium channels. *Proc. Natl. Acad. Sci.* **109:** 3558–3563

St Onge RP, Mani R, Oh J, Proctor M, Fung E, Davis RW, Nislow C, Roth FP & Giaever G (2007) Systematic pathway analysis using high-resolution fitness profiling of combinatorial gene deletions. *Nat. Genet.* **39:** 199–206

Su Y-F, Yang T, Huang H, Liu LF & Hwang J (2012) Phosphorylation of Ubc9 by Cdk1 Enhances SUMOylation Activity. *PLOS ONE* **7:** e34250

Timm DE, Liu J, Baker L-J & Harris RA (2001) Crystal structure of thiamin pyrophosphokinase1. *J. Mol. Biol.* **310:** 195–204
